# Supplementary material for: The fall armyworm strain associated with most rice, millet, and pasture infestations in the Western Hemisphere is rare or absent in Ghana and Togo
Source: PLoS One. 2021 Jun 21;16(6):e0253528. doi: 10.1371/journal.pone.0253528 (PMC8216543; doi:10.1371/journal.pone.0253528)
Supplement: Attachment — Submitted filename: suggestion.pdf [file pone.0253528.s001.pdf]

In this paper, authors described “The fall armyworm strain associated with most rice, millet, and pasture infestations in the Western Hemisphere is rare or absent in Ghana and Togo.” Authors have field detection and lab hybrid work upon FAW. Data providing was sufficiently enough, while the inconsistent or conflict recognition on R-strain or C-strain confuse their result and the following discussion.

Actually, authors have a conflict result upon the FAW recognition of R-strain and C-strain between the mitochondrial and nuclear DNA markers, i.e. intermediate types of COI-CS/TpiC and COI-RS/TpiC. They also mentioned and suggested that the COI gene, is not an accurate indicator of strain identity for the African fall armyworm populations. Therefore, how do they elucidate definitely the R-strain is rare or absent in Africa.

Several molecular evidences have shown that R- and C-strains with undistinguishable morphology are generally sympatric in the field, implying the possibility of genetic exchange between the two evolutionary groups. Moreover, molecular evidences also revealed that more than two genetically distinct clusters existed in FAW; therefore, distinguishing the C-strain from Rice-form based on their host plant and genetic markers is unlikely necessary. Authors also shown African FAW do not display strain-specific mating patterns.

It is undoubtedly important work to know the population structure upon African FAW, but it documented unsuitable based on R- and C-strain.
