# Supplementary material for: The fall armyworm strain associated with most rice, millet, and pasture infestations in the Western Hemisphere is rare or absent in Ghana and Togo
Source: PLoS One. 2021 Jun 21;16(6):e0253528. doi: 10.1371/journal.pone.0253528 (PMC8216543; doi:10.1371/journal.pone.0253528)
Supplement: Attachment — Submitted filename: suggestion for R1.pdf [file pone.0253528.s003.pdf]

Authors could have an outstanding elucidation for African FAW structure, while the R-strain and C-strain is now not a natural recognition.

As the authors showed either in the previous version or this version, L71-79, that hybridization between Corn-strain and Rice-strain has been occurred and showed disagreement between the mt*COI* and nr*Tpi* strain haplotypes. Authors also know that there have several publishings documented that more than two genetic lineages could be found in FAW. It is already unmeaningful to distinguish R- from C-strains for FAW as I have suggested in the previous version. It is undoubtedly important work to know the population structure upon African FAW, but the R- and C-strain documentation should be abandoned.
