# Supplementary material for: The fall armyworm strain associated with most rice, millet, and pasture infestations in the Western Hemisphere is rare or absent in Ghana and Togo
Source: PLoS One. 2021 Jun 21;16(6):e0253528. doi: 10.1371/journal.pone.0253528 (PMC8216543; doi:10.1371/journal.pone.0253528)
Supplement: Attachment — Submitted filename: Response to Reviewers.docx [file pone.0253528.s002.docx]

Response to reviewers.

We thank the reviewers for their comments and have tried to address all the concerns. Extensive changes were made to the introduction and discussion to provide a clearer explanation of the strains and markers, as well as the rationale for the experiments. Minor alterations were made to Figures 5 and 6 that did not change the conclusions made from the data. Reviewer’s comments are bracketed (< >).

<1. As the authors already showed in their previous papers, the vast majority of fall armyworms in Africa are hybrids between corn and rice strains… Thus, what we know now is that fall armyworms from corn field in invasive populations are hybrids, not corn strain.>

The assertion that the Africa fall armyworm are hybrids is an explanation for their unusual configuration of genetic markers. However, there is no indication that they are hybrid in their strain identity (in other words as far as we can tell the African fall armyworm are behaving like the C-strain in their plant host use). A paragraph was added to the introduction (lines 71-79) to explain this distinction.

<But throughout the whole manuscript, it appears that they assume that African fall armyworms from corn fields are corn strains. But this assumption seems to be incorrect.>

We have gone through the manuscript to remove such assumptions. For example, the term C-strain or corn strain are not used in the Results section. Instead, the specimens are described by their COI or Tpi haplotype. We also now provide in the discussion an explanation of why we believe the African fall armyworms can be considered C-strain (lines 375-385)


<2. In the same context, they have to make it clear what they mean corn strain and rice strain. Are they a taxonomic group? Or just grouping according to the sampling site?>

We added a paragraph to the introduction describing the difficulties in categorizing what the strains represent (lines 47-55). In addition, we note that lines 56-70 in the introduction describe method and rationale for how we defined strains in this paper.

<3. Line 51-52. Independent segregation between CO1 and TPI is not true. A half of nuclear genomes is from mother. Thus, 50% of genomes co-segregate with mt-CO1.>

We corrected this error in lines 60-62.

<4. Table 1, to me, larvae collected from plants by hand could be very different from adults collected by pheromone trap. The adults collected near rice field might have grown up in corn field. They need to show that these adults were grown up on rice field in order not to say that corn strains accidentally arrived near rice fields.>

As noted in lines 184-190, the larval collections are treated differently from pheromone trap collections when appropriate. We discuss at some length the possibility that the rice pheromone trap specimens come from other hosts (lines 360-372) and have tempered our conclusions with that in mind. We added references to examples where pheromone traps in the Western Hemisphere have successfully collected populations with strain identities that correspond with the local host plants (lines 354-355).

<5. L229-L230. They have to provide a reference. And it is really unclear because, at L23-L25, they say that fall armyworms are not a major risk for rice.>

We don’t believe a reference here is unnecessary since in this section we describe in detail fall armyworm infestations of rice in South America (references are in 243-247). We also removed lines 23-25 from the revised abstract.

<6. L347. Indeed, they analyzed a very low number of samples from rice field. Thus, all statistical analyses could not be performed properly because of insufficient statistical power. I do understand that it could have been difficult to collect insects from rice field. And this fact itself might be sufficient to say that there is no established population on rice field.>

We do not understand this criticism. If we look at Figure 5 for example the sample size for rice ranged from 45 to 109, which is pretty high for this type of study and similar in range to the sampling from corn.

Reviewer #2: In this paper, authors described “The fall armyworm strain associated with most rice, millet, and pasture infestations in the Western Hemisphere is rare or absent in Ghana and Togo.” Authors have field detection and lab hybrid work upon FAW. Data providing was sufficiently enough, while the inconsistent or conflict recognition on R-strain or C-strain confuse their result and the following discussion.

We made major revisions to the introduction and discussion to address this criticism.

Actually, authors have a conflict result upon the FAW recognition of R-strain and C-strain between the mitochondrial and nuclear DNA markers, i.e. intermediate types of COI-CS/TpiC and COI-RS/TpiC. They also mentioned and suggested that the COI gene, is not an accurate indicator of strain identity for the African fall armyworm populations. Therefore, how do they elucidate definitely the R-strain is rare or absent in Africa.

The conclusion that the R-strain is rare is based on the frequency of the TpiR marker and lack of fall armyworm in host plants and habitats preferred by the R-strain. In response to the reviewer, we expanded the relevant section of the Discussion on this point (lines 349-374). We also describe why we believe that the Tpi marker may still be relevant for strain identification in the introduction (lines 71-79) and Discussion (lines 375-399).

Several molecular evidences have shown that R- and C-strains with undistinguishable morphology are generally sympatric in the field, implying the possibility of genetic exchange between the two evolutionary groups. Moreover, molecular evidences also revealed that more than two genetically distinct clusters existed in FAW; therefore, distinguishing the C-strain from Rice-form based on their host plant and genetic markers is unlikely necessary.

Not sure what other genetic clusters are being referred to here, but we believe the strains are of particular importance because they differentiate fall armyworm based on plant host use. This has important implication when determining what plant types are at risk. We added a section to better explain this point of view to the introduction (lines 71-86). We also added additional material to the discussion describing our interpretation of the fall armyworm strains found in Africa (lines 375-399).

Authors also shown African FAW do not display strain-specific mating patterns.
It is undoubtedly important work to know the population structure upon African FAW, but it documented unsuitable based on R- and C-strain.

We disagree. At this time the strain markers are the only ones that have been demonstrated (in the Americas) to distinguish fall armyworm for their host plant preference, which has obvious relevance to identifying what crops are at risk. As detailed above, we have made extensive revisions to the introduction and discussion to explain our perspective.
