# Supplementary material for: The fall armyworm strain associated with most rice, millet, and pasture infestations in the Western Hemisphere is rare or absent in Ghana and Togo
Source: PLoS One. 2021 Jun 21;16(6):e0253528. doi: 10.1371/journal.pone.0253528 (PMC8216543; doi:10.1371/journal.pone.0253528)
Supplement: Attachment — Submitted filename: Response to Reviewers2.docx [file pone.0253528.s004.docx]

We do not agree with the reviewer’s comments.

The primary objection of the reviewer is: “It is already unmeaningful to distinguish R- from C-strains for FAW as I have suggested in the previous version”. We strongly disagree with that statement and believe the reviewer has not taken into consideration the following points.

First, all previous work on FAW population genetics in Africa have been done on specimens collected from corn or less frequently sorghum. These are host plants associated with the C-strain. Very little is known about whether FAW is associated with R-strain hosts in Africa and if so, what the strain composition of these might be. I want to emphasize that point, the characterization of FAW associated with R-strain host plants in Africa has not been done previously and without that analysis one cannot assume the absence of the R-strain in Africa. To do so, as appears to be what is being suggested by the reviewer, is not good science. This point is explicitly made several times in the paper (see for example lines 8-16 in the abstract, lines 85-89 in the introduction, and lines 353-354 in the Discussion). It is even alluded to in the lines noted by the reviewer (L71-79) where we state “Because virtually all the Eastern Hemisphere specimens tested to date were collected from the C-strain associated hosts corn and sorghum,…” (L74-76).

This is the issue being addressed by this manuscript. This is the first, and so far only, attempt to systematically collect and genetically analyze FAW in Africa from R-strain hosts. As such we believe it is a critically important paper for assessing the status of the two strains in Africa. To make this point explicitly clear lines 79-81 and 88-89 were added to the Introduction.

Second, the assertion by the reviewer that the strains are no longer relevant in Africa and that

“the R- and C-strain documentation should be abandoned” is at best premature and most likely wrong. The defining characteristic of the strains is host plant preference. The defining characteristic of the C-strain is that it primarily infests corn and sorghum fields, only occasionally rice, and very rarely pasture grasses. This manuscript demonstrates that the Africa FAW is showing a similar preference pattern. It is found in corn, is ambiguous in rice (trap captures but little evidence of larval infestation), and virtually absent in pasture grasses. So even if there was an ancestral hybridization event between the strains, the Africa FAW population is effectively still behaving like the C-strain. What has changed is that COI is no longer an accurate strain marker. This point is explicitly made in Lines 74-78 in the Introduction and 384-387 in the Discussion. We also provided an explanation for how this might have occurred where an early hybridization event between strains disassociated the COI marker but still maintained a C-strain identity/behavior (lines 377-401).

Third, the reviewer does not consider the fact that the R-strain does exist in the Western Hemisphere and so could enter Africa at any time. So even if the current African FAW is a hybrid, secondary introductions of FAW to Africa could return both strains into the continent with potential economic consequences. Therefore, continued surveillance of Africa populations with the strain markers is still relevant and important as is noted in lines 421-424.

The reviewer also stated: “Authors also know that there have several publishings (sic) documented that more than two genetic lineages could be found in FAW.” We believe these lineages are irrelevant to this paper as the lineages are not associated with host preference. This paper is about whether the two FAW lineages associated with different host plant preferences in the Western Hemisphere are present in Africa.

In summary, we believe the reviewer’s objections are not reasonable and stem from assumptions that are premature and probably incorrect. This manuscript represents the first systematic attempt to find and genetically characterize Africa FAW in the R-strain hosts of rice and pasture grasses. This information is critical to the assessment as to whether the fall armyworm population likely to threaten rice, millet, and pasture grasses is present Africa.
